# Barriers and facilitators to injectable opioid agonist treatment engagement within a structural vulnerability context: a qualitative study of patient experiences in Vancouver, Canada

**DOI:** 10.1186/s12954-025-01262-4

**Published:** 2025-07-04

**Authors:** Samara Mayer, Nadia Fairbairn, Al Fowler, Jade Boyd, Thomas Kerr, Ryan McNeil

**Affiliations:** 1 Institute on Aging and Lifelong Health, University of Victoria, 3800 Finnerty Road, Victoria, BC Canada V8P 5C2; 2 Canadian Institute for Substance Use Research, University of Victoria, 3800 Finnerty Road, Victoria, BC Canada V8P 5C2; 3 BC Centre on Substance Use, 1045 Howe Street Suite 400, Vancouver, BC Canada V6Z 2A9; 4 Department of Medicine, University of British Columbia, St. Paul’s Hospital, 608-1081 Burrard Street, Vancouver, BC Canada V6Z 1Y6; 5 Internal Medicine, Yale School of Medicine, New Haven, USA 06510; 6 Yale Program in Addiction Medicine, Yale School of Medicine, New Haven, USA 06510; 7 Department of Anthropology, Yale University, New Haven, USA 06510

**Keywords:** Injectable opioid agonist treatment, Qualitative, Ethnography, Structural vulnerability, Patient engagement

## Abstract

**Background:**

Amidst a sustained drug poisoning crisis, there is growing recognition in Canada of the need to expand injectable opioid agonist treatment (iOAT). iOAT is an intensive treatment that involves the daily self-administration of hydromorphone or diacetylmorphine under healthcare provider supervision, typically accompanied by other health and social services. While this treatment has demonstrated effectiveness in reducing drug use-related risks, high threshold characteristics may also create barriers to engagement. This study examined patients’ experiences of barriers and facilitators to iOAT with attention to how social and structural factors (e.g., housing vulnerability, poverty) shape program engagement.

**Methods:**

This study draws on qualitative interviews and fieldwork observations with people accessing four iOAT programs in Vancouver’s Downtown Eastside neighbourhood from May 2018 to November 2019. Data included baseline and follow-up interviews and approximately 50 h of observational fieldwork. Analysis leveraged a structural vulnerability lens to examine how social and structural factors shape people’s engagement with iOAT.

**Results:**

Participants highlighted how improved access to health and social services, compassionate and relational care, and flexible and individualized approaches to treatment delivery that addresses and accounts for the structural vulnerabilities facilitated engagement in treatment. However, dosing supervision, operational capacity and medication formulation were experienced as barriers to treatment. These barriers were magnified by structural vulnerabilities such as housing instability and mobility challenges.

**Conclusions:**

Study findings highlight how people navigate the barriers and facilitators to iOAT engagement in light of the structural vulnerabilities they experience. Adaptations to and ongoing support for iOAT programs may help to facilitate engagement and should focus on equity-oriented and patient- centered treatment models that includes the integration of social supports, support for relational care and treatment planning that supports patient autonomy.

## Introduction

Canada and the United States are experiencing an ongoing overdose crisis that has reduced or slowed life expectancy gains [1]. This crisis is fueled by a highly toxic drug supply, with fentanyl and fentanyl-related analogues, and increasingly benzodiazepine adulterated fentanyl playing a central role [2]. Between 2016 and March 2024, more than 45,000 people in Canada died from apparent opioid toxicity [3] and 81% of overdose deaths between January and March 2024 were linked to fentanyl and related analogues [3]. The overdose crisis is also deeply intertwined with broader social inequities, such as poverty and unstable housing, and there has been a disproportionate impact of this crisis on Indigenous, Black, and Hispanic communities [4–6].

As part of the response to the overdose crisis in an effort better support people who inject unregulated and criminalized drugs who might not have benefitted from other treatment options (e.g., buprenorphine, methadone), injectable opioid agonist treatment (iOAT) has gained attention [3, 7, 8]. In Canada, iOAT mainly involves the use of injectable hydromorphone (HDM) and, to a lesser extent, diacetylmorphine (DAM), also known as heroin assisted treatment [9]. To qualify for this treatment, individuals must have a history of opioid injection use, current injection drug use, have a diagnosis of severe opioid use disorder and the capacity to consent to treatment [9]. This intensive treatment involves daily injections under healthcare supervision (typically nurses), often alongside additional primary health and social services [9]. In March 2019, there were 405–420 patient places across 12 programs nationally, however many jurisdictions, including small and suburban communities and Canada’s most populous city, Toronto, had no iOAT services [10].

While iOAT programs are not widely available in Canada, iOAT programs are supported and informed by robust randomized clinical trial data from studies conducted in Europe and Canada [11–13]. Meta-analyses of these studies have shown that among individuals who did not benefit from methadone, DAM administered under the supervision of trained health professionals in a clinic setting is beneficial in terms of reducing illicit opioid use, premature treatment discontinuation, engagement in criminalized activity, incarceration, and mortality, as well as improving overall health [11, 12]. Qualitative research on clients’ experiences of iOAT in clinical trial and non-clinical trial settings have highlighted tensions between the regulatory constraints of treatment and treatment intensity and the positive outcomes associated with supportive treatment, and having access to an injectable opioid treatment option [14–17]. In Canada, significant efforts have been made to understand and improve client experiences in post clinical trial settings, including a focus on how to implement and optimize patient-centered care practices and optimize dose satisfaction [17, 18].

Patients who access iOAT are often significantly impacted by social and structural inequities (e.g., poverty, health inequities, stigma), which can impact their treatment experiences. For example, approximately 60% of people who participated in a clinical trial of this treatment in Canada were unstably housed (including single room occupancy housing with restrictions) or homeless (living in vehicles, public places) in the month prior to the study [13]. Moreover, approximately one-third of participants self-identified as Indigenous and presented to treatment with greater experiences of discrimination and resultant social inequities (e.g., lower educational attainment, higher rates of foster care) compared to non-Indigenous participants [19]. Research has indicated that iOAT can help to improve the health outcomes of people who experience social inequities [13, 15, 19] and the application of patient-centred care approaches in iOAT has been found to help overcome prior experiences of stigma [20]. As iOAT is implemented in “real world” settings there is a need to continue to examine how intersecting social inequities and disparities might impact treatment experiences and how treatment programs address and account for such inequities. This study therefore examined patients’ experiences of barriers and facilitators to iOAT within a structural vulnerability context.

## Study context and recruitment sites

This study was conducted in Vancouver, Canada’s Downtown Eastside, a neighbourhood heavily impacted by structural marginalization including, poverty, housing insecurity, gendered violence, and the criminalization of drug use, particularly among Indigenous peoples [21–24]. The neighbourhood has a higher concentration of harm reduction services including, Canada’s first supervised injection site (Insite), overdose prevention sites, safer supply programs, and addiction clinics [25, 26]. Longstanding community advocacy has played a central role in shaping drug policy and expanding access to services in the neighbourhood, including iOAT programs [27–29]. iOAT started in Vancouver with randomized clinical trials including the 2005 North American Opiate Medication Initiative (NAOMI) and 2011 Study to Assess Long-Term Opioid Medication Effectiveness (SALOME) which demonstrated the efficacy of DAM and HDM [13, 30]. Despite strong evidence, regulatory and political resistance delayed widespread implementation of this treatment [29]. Initially available only in one setting in Canada, the site of two randomized control trials of this treatment, iOAT has since modestly expanded after 2016 due to increased funding and regulatory changes as part of the response to the overdose crisis [10].

Participants in the study were recruited from four clinical programs that provide iOAT located in Vancouver’s Downtown Eastside, including: (1) a program within a harm reduction facility featuring an overdose prevention site, linked to a nearby off-site health clinic operated by a non-profit; (2) a pharmacy-based program; (3) a program integrated into a healthcare clinic with an in-house pharmacy; and (4) a comprehensive program that also served as the site for the previous randomized controlled trials. Patients receive up to 3 daily injectable doses of HDM or DAM, depending on the clinical model, and receive concurrent oral therapies such as slow-release oral morphine and methadone. These programs do not generally allow people to receive take-home doses or “carries” for injectable HDM/DAM or concurrent oral therapies (e.g., methadone, slow-release oral morphine) [9]. Programs do not permit health care providers to provide intra-venous injection of HDM or DAM. However, nurses can provide intra-muscular injection. The programs have differences in terms of service integration, operational models, and the types of medications offered (described in further detail in Table 1). While there has been policy changes [31] and guidance [9] to support the delivery of DAM, this medication remains highly regulated and is more expensive. In contrast, HDM is less regulated and more affordable and therefore more widely available [10, 31].

**Table 1:** Characteristics of iOAT Sites

	Model one	Model two^a^	Model three	Model four
Service delivery model	Embedded and integrated I; overdose prevention site—separate entrance and injection space	Pharmacy-based	Embedded and integrated II; community health centre—shared entrance, separate injection space	Comprehensive and dedicated standalone model
Onsite staff	Nurses, mental health workers, peer support workers	Nurses, peer support workers, pharmacists, pharmacist technicians	Nurses, physicians, nurse practitioners, community liaison workers, pharmacists, pharmacist technicians	Nurses, clinic assistants
Hours of operation for medication access	7	6.75	7	13.5
Available iOAT medications	HDM	HDM	HDM	HDM/DAM
Number of available daily doses	2	2	2	3
Total number of active patients (Sept 2018)	Data not available	Data not available	4	126 (DAM = 106)
Total number of active patients (2019)	Data not available	Data not available	11	125 (DAM = 107)
Program capacity (Sept 2018)	30	65	14	130–145
Program capacity (Mar 2019)	60	–	14	130–145

Information about each of the service models in this study was also collected as part of an environment scan conducted at the same time as this study (2018–2019) [10]

^a^In 2019, the pharmacy-based program (Model 2) ended, and participants transitioned to Model one

## Methods

### Conceptual framework: structural vulnerability

This study leveraged the concept of structural vulnerability as a conceptual framework. This concept refers to the way that social, economic, and political systems create and sustain disparities in health and well-being across different groups of people [32]. It emphasizes that some populations are more likely to experience poor health outcomes due to their position within broader structures of power and inequality that often result in unequal access to resources, services, and opportunities, including health care [32]. Structural vulnerability was used in this study to analyze the pathways that shape inequities in care and health within iOAT programs. This lens provided a way to examine how iOAT programs adopt approaches responsive to the structural vulnerabilities that people experience in their lives (e.g., providing access to health services, connections to housing, providing culturally competent care) and how programs may have the inverse effect of reflecting structural vulnerabilities, reinforcing health inequities, and even negatively impacting care outcomes [33].

### Data collection

This longitudinal qualitative study included baseline interviews, one-year follow up interviews and ethnographic observations. SM and AF collected data. SM is a health researcher and AF has experience with various drug treatment program (e.g., iOAT, methadone, safe supply), and has ties to Vancouver-based drug user advocacy groups. A total of 52 participants were recruited during site visits by direct referral from iOAT care providers (e.g., nurses, mental health workers, and peer health navigators), during ethnographic observations or were recruited from a parallel quantitative iOAT cohort study that was implemented at the same time as this qualitative study. An effort was made to recruit participants initiating treatment to follow their trajectories and experiences over time to better understand how their experiences evolved and to overcome potential recall bias and to recruit diverse participants for interviews based on their demographic characteristics. This included oversampling for women, younger people (23–66 years of age), Indigenous people and non-Indigenous people of colour to provide more diverse representation and enable analyses of how people with different social identities experience iOAT. Recruitment for baseline interviews took place from May 2018 to November 2019 (ending due to COVID-19) and resulted in 52 participants across various treatment models.

Interviews were guided by a semi-structured interview guide developed and refined by the research team and a community advisory board (CAB). The CAB included people with experience recieving iOAT from different programs in Vancouver. The CAB met with the research team throughout the study to collaborate on implementation, including data collection materials and recruitment approaches. Interviews were audio-recorded and transcribed by a third-party transcription service. Fieldnotes were also taken after each interview session to inform future data collection and analysis [34]. Ethnographic fieldwork observations involved direct engagement with iOAT participants in both clinic settings and community spaces. Fieldwork focused on understanding participants' everyday contexts and relationships [35], with 50 h of observation conducted. Ethnographic fieldwork initially took place at one iOAT program, involving informal conversations with participants in the waiting area or injection room if they provided verbal consent. Due to space restrictions in 2019, fieldwork shifted to community spaces with three participants, which involved informal conversations between the researchers in spaces of the participant’s choosing. These participants consented to participate in fieldwork during their qualitative interview and were asked to participate in field work based on the rapport developed between researchers and participants.

Follow-up interviews were conducted beginning at 12 months in 2019 with 19 participants from the integrated and pharmacy-based models (out of the 29 participants who were due for 1-year follow up interviews). In March 2020, additional planned baseline and remaining follow-up interviews, and ethnographic fieldwork were suspended due to COVID-19. While remote data collection was attempted, implementation faced logistical and ethical challenges as the health care system, including iOAT programs, were strained due to the pandemic and many community members tragically died due to the worsening of the toxic illicit drug supply. By November 2020, data collection was permanently suspended due to these barriers. As a result, follow-up interviews and additional baseline recruitment from model 4 (see Table 1) were not completed. This study received approval from UBC/PHC research ethics board, H17-00557.

### Data analysis approach

Initial descriptive coding [36] was used to identify broad themes that focused on operational aspects of iOAT programs that were considered facilitators and barriers to the program, this included categories such as care providers and program operations. After establishing these descriptive categories, the data set was then reviewed with attention to how social factors impacted participants’ experience of iOAT (e.g., housing, income generation activities, stigma, racism, incarceration). In applying the conceptual framework of structural vulnerability, the broad operational categories and the social categories were considered together to refine the themes. These themes were presented to the iOAT CAB for feedback who emphasized results that they felt were particularly important to report, including the importance of social support, relational qualities of iOAT and the need to better support and improve the capacity of programs. Participants were assigned pseudonyms using a random online name generator. Due to COVID-19 related barriers, there was not enough data to conduct in-depth comparative analysis between the iOAT models over time. Despite these challenges, efforts to maintain community engagement continued through iOAT CAB meetings, although changes in members' life circumstances and the shift to remote meetings led to lost contact with several members.

## Results

Findings present participants experiences and perceptions of facilitators and barriers to iOAT within a structural vulnerability context. The demographic characteristics of patients who participated in baseline and follow up interviews are presented in Table 2.

**Table 2:** Demographic characteristics of participants

Participant characteristics	Baseline n = 52 (%)	Follow-up n = 19 (%)
*Age*		
Mean	45	44
Range	23–66	28–57
*Gender*		
Man	36 (69%)	14 (74%)
Woman	16 (31%)	5 (26%)
*Ethnicity*		
White	30 (58%)	11 (58%)
Indigenous	21 (40%)	8 (42%)
Did not wish to disclose	1 (2%)	0 (0%)
*Type of housing*		
SRO—public	19 (37%)	9 (47%)
Apartment	10 (19%)	5 (26%)
Unsheltered/Outside	8 (15%)	3 16%)
Shelter	7 (13)	0 (0%)
SRO—private	5 (10%)	4 (21%)
Friend’s place	2 (4%)	0 (0%)
Detox	1 (2%)	0 (0%)
*Income generation in the last 30 days*		
Social assistance—income support, disability	47 (90%)	16 (84%)
Part-time work^a^	19 (37%)	7 (37%)
Drug selling	17 (33%)	5 (26%)
Vending/re-selling goods on the street or at markets	14 (27%)	6 (32%)
Recycling	16 (31%)	4 (21%)
Boosting/theft)	12 (23%)	3 (16%)
Panhandling	12 (23%)	2 (11%)
Sex work	4 (8%)	1 (5%)
Full-time work	1 (2%)	0 (0%)

^a^Includes work at building and peer roles at overdose prevention sites and iOAT programs

### Facilitators

#### Improved access to health and social services

Through iOAT, patients were able to receive other medications in addition to their HDM or DAM, such as anti-depressants, antiretroviral, and direct-acting antiviral medications. They could also receive other health services including primary care, wound care (e.g., bandages, cleaning, and antibiotics), and referrals to health services and specialists (e.g., cardiologists). Participants—particularly those who had chronic and/or complex medical conditions or were older—found that iOAT improved their access to health care. Some participants described that prior to iOAT they had not regularly engaged with health services. For example, Andrew highlighted:

*I’ve never gone to the hospital before, so this is the gist of the healthcare that I’ve gotten in my life […] Yeah, if I actually had a serious health problem, I would have somewhere that is not daunting for me to go to. I could just go take care of some shit quickly instead of it being like before, it’s like what do I even do? (29-year-old Indigenous man)*

Here, the participant described how being engaged with iOAT helped to remove barriers to health care allowing patients to address health concerns and address health issues earlier and avoid health crises. This enabled a positive feedback loop in that some patients were able to better engage with the iOAT program because their general health improved. Other participants similarly remarked that having integrated health care enabled providers to have a more comprehensive understanding of their health. As Daniel explained:

*Because you want one doctor to… like they’re handling other issues for me as well. Like, I have some stuff going on with my stomach, and they’re helping me with that. They know what’s going on with me, complete package, right? Not just methadone doctor, another doctor, and another doctor, right? Like they know my whole thing, right? And they handle my whole thing, so I like that. (34-year-old white man)*

As this participant highlights, through iOAT their health and substance use-related needs were approached holistically. Moreover, as part of an integrated care approach, some iOAT programs also helped to coordinate patient care. For example, some participants noted that iOAT providers would remind them of other health care appointments, help to arrange transportation to these appointments and/or sometimes accompany patients to different health care services. These activities were directly responsive to the needs of patients who had mobility barriers, had memory loss issues, and/or who found it challenging to make appointments due to insecure housing or ongoing illicit drug use. As highlighted by Amy:

*They actually help you go to your appointments; you know. That’s awesome. Like you know, but in the beginning, they didn’t have any of that stuff because it was the study. (46-year-old white woman)*

Here, the participant describes the benefits of additional provider support which was previously not as available when iOAT was provided as part of a clinical study. Lastly, some iOAT programs were embedded in organizations that offered housing and employment opportunities, offering patients a network of supportive services. These programs offered peer worker programs, provided housing support and helped patients to access their social assistance payments. However, not all iOAT programs were able to offer this network of services and support.

#### Compassionate and relational care

Aligning with other research on iOAT [14, 17], participants highlighted the importance and centrality of having supportive relationships with health care providers. Participants reported that providers treated them like “human beings” and that they were “non-judgemental” and “seemed to genuinely want to help”. These relationships were notable because many participants had longstanding histories of negative encounters with the health care system, including, for instance, MMT programs and hospitals. As highlighted by John:

*Yeah, you know what? [Laughs] I have no idea what they think inside their heads, right? But, you know, it really seems like they genuinely want to help, and that’s what I’ve always wanted to have. It’s just someone to say, “Hey, I care enough about you to care about you until you can care about yourself,” right? And it’s a nice feeling. (41-year-old Indigenous man)*

Other participants similar emphasized that providers felt like “family” or “friends” and participants thus felt cared for as “whole people”. Participants valued having time to talk to care providers about their health, care goals and what was going on in their lives. For example, Josh had experienced a traumatic family situation which caused him to increase his illicit drug use:

*I noticed my usage was going up. But I talked to [care provider name] at the clinic about it, and I just brought it up and said, “This is what’s happening. My usage is going up,” and stuff like that. And they just kind of checked in with me every day after that, to see how I was doing and stuff. And eventually the feeling, I got over it, you know. (37-year-old white man)*

Here, Josh described how having a therapeutic relationship with a care provider meant that he could receive emotional and social support in ways that helped him to stay engaged with treatment during a challenging time in his life. Notably, the care provider took time to check in with him daily about how he was feeling. Other participants similarly described the importance of providers taking time to connect with patients and some reflected on how the ability to stay in the iOAT program before or after receiving their medication to watch TV, have coffee, or just sit gave them more time for connection. This was particularly valuable for people experiencing housing insecurity because it provided them a safe space to rest. Some programs had peer support workers (where lived experience of substance use is identified and central to their role) that participants felt was useful because it helped reduce the strain on other care providers and they felt it was easier to relate to care providers with shared experiences of drug use. As explained by Jason regarding his interactions with peer workers:

*Somebody who’s working there [nurse, social workers ect.] doesn’t understand what’s going on right there, right? They’re, “Come on, let’s go. You’ve got this much time. You’ve got this much time.” The peers understand it a little bit more, because what’s going on, right, and you can’t find your veins or something…If something happens, we can always read each other. Always. Like you don’t have to say much. We can just read each other, right? (37-year-old white man)*

Here the participant described how some programs have time limits to take their injection to reduce wait-times for other patients. Time limits was described as stressful for some patients who were unable to find a vein, however, as this participant notes, peer workers helped because they could connect with patients based on shared experiences. While providers were not permitted to directly assist with IV injection, care providers would sometimes help patients indirectly by, for instance, moving lamps closer or bringing them a warm glove to dilate their veins. Some peer workers interviewed, who were also iOAT patients, reported that they provided emotional support for patients by taking time to sit and talk to patients and some peers also tried to facilitate connections to housing and employment for patients.

#### Flexible and individualized approaches to treatment

Flexible and individualized approaches to treatment included when treatment was open ended and the small changes providers made to program operations to accommodate patient’s social and physical needs. Open-ended treatment meant that patients could re-access the treatment as often as needed and would not be cut-off treatment due to missed attendance or periods of disengagement, which some participants had experienced with other treatment types. Some participants experienced insecure housing, were hospitalized for prolonged periods, or went through periods of incarceration and, therefore, missed receiving their treatment for a few days or even months. As described by Andrew when asked about his experience returning to iOAT after being in jail:

*It was fine. Everyone there was great. There is one [care provider] there, apparently, she was really nice though about like, I would be coming out of jail and I’d be like oh fuck, and I’d be like let’s go over there and see if we can work something out, and she’d always be like yeah, come. She wasn’t technically supposed to be doing it but she recognized me and just put me right back on. (29-year-old Indigenous man)*

As described in this quote, Andrew felt that it was because the care provider knew him that he was able to re-enter treatment easily. Other patients similarly reported that they felt that missed treatment attendance was not treated in a punitive way and felt comfortable re-accessing treatment as often as needed. As described by Ryan when asked about why he stopped iOAT:

*It just wasn’t working out. I just can’t remember. I was using [illicit drugs], I was taking methadose, like it was just too much; I couldn’t manage it all really. One thing though that they had told me is that if you ever want to get back on it, you can see a doctor, you talk about it and see if it, you know, see if again, if you’re eligible. That’s what I did last Friday. I came in and talked about it. Just trying to figure out why it didn’t work, and why I think it would work now. And if my state of mind was really good for it, or if I’m just going to quit again. And sure enough, I stuck it out. I'm not going back to Methadose. So, I’m trying to stick with this. (34-year-old white man)*

Here, the participant emphasizes how being able to return to the treatment when he was ready allowed him to better engage in iOAT. Another approach to flexible treatment included modifications to program operations such as: allowing patients to be a little late for their shots, giving individuals more time to take their shots and facilitating more rapid dose titration schedules due to higher opioid tolerance from the toxic drug supply after periods of treatment disengagement. However, some participants noted that the ability of providers to make these changes was dependent on the therapeutic relationship they had with the provider, including the knowledge the provider had of the participant’s social context (e.g., housing) and ongoing drug use that allowed them to make determinations of the safety of more aggressive dose titrations and avoid potentially adverse medication reactions. These changes were also dependent on the capacity of the program and the power that the provider had to make these changes. Indeed, some participants reflected on how some care providers were stricter in enforcing rules, whereas other care providers, generally those that participants’ felt had worked at the iOAT program longer and/or who the participant had developed a stronger relationship with, were more flexible.

The value of highly individualized approaches was also made evident in ethnographic fieldwork with a participant who experienced significant adverse reactions to HDM and who experienced housing insecurity and drug debts in the neighbourhood where the clinic was located that made it hard for him to make it to the clinic consistently. In fieldwork and in interviews he noted that one of long-standing nurses at the program provided him individualized care that supported his engagement in iOAT. He noted that because she understood the challenges he faced in accessing treatment, she adapted the treatment and allowed him to take more time to take his shot, broke up his shot into two doses so it was easier to inject and as he noted, took his concerns seriously.

### Barriers

#### Dosing supervision and operational capacity

The most commonly reported challenge to engaging with iOAT was the daily witnessed dosing requirements (1–3 times per day). Daily witnessed dosing was challenging for all participants, but particularly so for patients who worked or wanted to work, were sick, had mobility considerations, who wanted to connect with their family and/or who lived further away from the programs. As described by Amanda:

*I think they need to… they need to be aware that of people’s mobility issues and stuff like that, where because there’s certain people that can’t… can’t make it up there twice a day. You know what I mean? It’s hard for some people, like that are mobility-challenged, or even like my lungs. Like Sunday I had to call and ask them if they could send me a cab if they will, and they can’t do that that often. You know, and there should be a way to address that. Because I mean, it is… it is an issue. (38-year-old white woman)*

As this participant articulated, challenges in attending treatment daily could not consistently be addressed by care provider modifications. Additionally, participants described how daily witnessed dosing could create barriers to employment. As described by Kenneth:

*So yeah, then afternoon I go back to the clinic for another shot, and like my biggest problem, actually, my only criticism with the clinic is if I get a full-time job, they are not really flexible to accommodate you. If I have to be at work, let’s say, at eight o’clock, they don’t have your dose for seven o’clock. They’re just… they are not set up like that. So that’s the only problem. Like if someone wants to be productive and be able to go to work and get the treatment, they are not set up for that. They… like I went to this work agency, and they were helping me to find a job, and they found a bunch, and I go, “Well, I cannot do this and this, and this and this, because of the time… time schedules.” And they go, “Why?” “Well, because I have to go to a clinic.” And then they go, “What? Why? How?” So I tell them, and they go, “Well, sorry. Then you cannot do that. That’s impossible.” Yeah. (60-year-old white man).*

Here, Kenneth describes how daily witnessed dosing requirements can create barriers to employment, while some participants were able to adjust their work schedule to accommodate iOAT, others could not and were unable to work or would miss shots to accommodate their work schedule. Another participant described how they were unable to adjust their work schedule because they did not want to tell their employer they were part of an injectable drug treatment program as they were concerned about the stigma associated with this treatment. Participants similarly reported that hours of operation and program capacity could impact engagement. Most programs were open for 7 h a day between approximately 8AM and 4PM (with some closed over lunch). Some participants reported wanting the programs to be open later so that they could take doses in the evening so that they would better last overnight. As Tim described:

*If they could separate that time and spend more time in the afternoon, like do the nights so it stretches over the whole day, it would be nicer, instead of bunching it up in the morning and taking my overnight stuff, they give me morphine [Kadian] for my overnight stuff. (51-year-old Indigenous man)*

Participants further reported that sometimes in the mornings you had to wait for treatment because the program was at its maximum capacity, contributing to an overall increase in time spent at the program. As Mark describes:

*It’s alright but it’s very busy at the [iOAT program]. It’s very packed, especially certain times of the day, like first thing in the morning, uh, right before the end, 4 o’clock there’s a real rush and, uh, and they do their best but it’s, there’s a lot of people in a relatively small space so, uh, sometimes they have to wait, you know, to do my shot so it takes, it takes up a fairly significant portion of my time during the day, you know. It’s, you have to wait 15 minutes after you get your shot but a lot of times you have to wait 15 minutes before you even get your shot, you know, and then you have to do it and then you have to wait 15 minutes and then it’s twice a day and so it’s a bit time consuming but, uh, it’s still it’s better than the alternative. (54-year-old white man)*

This participant explained how wait times at the clinic increased the overall time he spent at the program, however as he, and other participants noted, the time expended at program was better than the alternative of accessing the illicit drug supply. Lastly, some participants described that generally in busier programs health care providers had less time to support and engage with patients*.* Other participants similarly reported that less busy programs enabled them to more easily communicate with care providers about their physical and mental health and treatment goals and as one participant noted “express themselves”. Further, in busier programs participants reflected on how they would prefer if the program was quieter and had less people so they would not get distracted and be able to wait inside on colder days which would help them better concentrate to be able to inject their medication and improve venous access because they could stay warm.

### Medication type

The potency of the illicit drug supply including the replacement of heroin by fentanyl and increasing toxicity of the supply could impact how participants experienced their opioid medications. Participants reported that increasingly high opioid tolerances made it hard for them to transition away from illicit opioids, as the medication available was not always able to help them avoid withdrawal symptoms, opioid cravings and/or pain. As described by Daniel:

*I think just my tolerance outweighed the amount that I could get, like once I got to that top amount where you can’t go any higher. Over time you’re going to need more and more, right. When that wasn’t available anymore to get more, then it started to get to the point where it was, I was getting sick. Yeah, I couldn’t get more and my tolerance was going higher. Yeah, I started using more because of the dope sickness. If I had money, I would not even go there and just use heroin. (34-year-old white man)*

Some participants felt that the medications were ineffective due to the HDM formulation and some felt it was due to insufficient dose strength. Those who felt the medication was ineffective due to the formulation were sometimes hesitant to increase their dose and were concerned about receiving high doses (described in further detail in [37]). Participants also had diverse opioid-related needs and experienced varying side-effects from HDM and DAM including rashes, pins and needles and a feeling of numbness in the appendage they injected it. Participants described how the negative side-effects of the medication could be overwhelming, negating the possible therapeutic benefits (e.g., pain relief, withdrawal management). Some participants also reported that it was challenging to inject the volume of HDM they were prescribed and felt they would benefit from having access to a wider variety of medication types and modes of administration to meet their needs. In describing their preference for a tablet HDM program [26], Anthony explains:

*I mean I don’t like the side effects. You know, from when I do find a vein, it’s very pins and needles. Like there’s… it’s like, you know, it kind of, just the way it hits you, right. It’s very obnoxious the way that hydromorphone hits you, right. It basically numbs your whole appendage, right. And you can just feel the damage it’s doing, right. Like I said, I much prefer the dilaudid just because you’re not shooting as much liquid. It’s just like the amounts of liquid that they’re making us inject is massive, right. Versus dilaudid, I mean I can cook those dilaudid down into one mil and have it filled up, you know, to the point six mark, right. I mean yeah. (53-year-old white man)*

Lastly, some participants reported that they would prefer access to DAM, over HDM, and referred to this treatment as “actual heroin”. However, they were unable to gain access to DAM as this treatment was only offered at one clinic that had a lengthy waitlist at the time of data collection. These participants described how they felt DAM would have the intended feeling they wanted because it was closer to heroin, their drug of choice, which they felt would improve their engagement in treatment.

## Discussion

Drawing on a structural vulnerability framework, this study highlights the structural, social, and economic facilitators and barriers to engagement in iOAT programs in Vancouver, Canada. Participants, particularly those with complex health needs and/or who experienced housing insecurity, described how access to a range of health and social services, helped them engage with treatment. Further, the relational care offered by iOAT care providers facilitated engagement as it enabled providers to be responsive to participant social contexts such as mobility challenges, including making small adjustments to program operations and ensuring participants were able to re-enter treatment. Challenges included the dosing supervision, operational capacity and medication formulation. Some participants felt that extended hours or less crowded environments would be responsive to mobility needs, employment obligations and would facilitate relational care. Additionally, the formulation of the prescribed medications, especially HDM with less access to DAM, were concerns for some, as high opioid tolerances and medication side effects impacted their effectiveness.

Overall, this study contributes to research that articulates a tension between the regulatory and the relational qualities of iOAT programs [14, 16, 17, 38, 39]. Research published in this study context has examined patient-centered care in iOAT, an approach to care that recognizes and supports people’s unique needs, values, and preferences through effective communication, partnership, and health promotion [40, 41]. This research has emphasized the centrality of building health care provider relationships for patient centered care to establish a foundation in which participants experienced care that was responsive to their individual dose, health and psychosocial needs [17]. Our study similarly emphasized how relational care facilitated flexibility and individualized treatment approaches that were attentive to shifting social context enabling treatment engagement. These findings further underscore the importance of identifying structural-level drivers that mediate the capacity of providers to deliver relational care and the need to support patient centered approaches to treatment delivery including for instance, care provider training and education, peer support worker roles, and health and wellness supports for care providers. In our study, structural factors, such as limited program capacity, crowded programs, hours of operation and high program demand, can impact patients’ experiences of relational and flexible care. Moreover, while open-ended treatment was identified as a facilitator to engagement, programs in Canada have long waitlists for treatment and availability is limited [10]. This may present a tension that programs have to navigate, that is, deciding whether or not to remove someone who is not attending treatment in order to bring in someone on the waitlist. Together these findings highlight the complexities that iOAT programs face in delivering relational and flexible treatment within the context of structural constraints that limit the capacity of drug treatment programs. As such, there is imperative to ensure the iOAT programs are adequately resourced to support care providers in providing relational care for patients.

This study’s focus on how structural vulnerabilities, such as housing insecurity, shaped patient engagement highlights the importance of incorporating equity-oriented approaches into iOAT. Equity-oriented care aims to reduce the effects of structural inequities, including the distribution of the determinants of health (income, housing), that sustain inequities and considers the impact of multiple and intersecting forms of racism, discrimination, and stigma on people’s access to services and their experiences of care [42]. Study findings suggest ways that iOAT can address and account for structural vulnerabilities by providing health and social services to meet patients’ needs. For example, some participants reported receiving health and social care at iOAT programs including, medication, health care, employment and housing support. Another study similarly explored the integration of iOAT at a comprehensive care site in Vancouver, examining its impact on service users and providers [43]. The study highlighted how such an approach to care can foster patient autonomy and supportive relationships, and a sense of place, including social connections and a community environment, suggesting that integrated care models can potentially strengthen treatment outcomes [43]. In this study, the provision of health and social supports helped patients engage with treatment, particularly those who experienced housing insecurity, and complex and chronic health conditions, suggesting that integrated programs that address social determinants are a crucial part of iOAT treatment delivery and equity-oriented care.

Service delivery implications from this study include a contribution to ongoing evidence about the value of establishing take-home doses in iOAT programs. In this study, daily witnessed dosing was commonly reported by participants to create challenges for treatment engagement. Previous research on methadone and buprenorphine has similarly documented strong preferences for take-home doses [44]. Moreover, other studies on MMT have found that dispensing policies, including daily witnessed dosing may create systemic barriers to employment for individuals enrolled in treatment [45]. This is because, similar to iOAT, the inability to obtain and consume methadone doses results in withdrawal symptoms and because this treatment is generally taken daily on a long-term basis [45]. Participants in this study similarly reported how daily supervised dosing policies can create barriers to employment that hindered their engagement in treatment.

In Canada, iOAT take-home doses were introduced after our study end date as part of public health measures implemented during the COVID-19 pandemic [46]. Later, some sites continued prescribing take-home doses for up to two of a possible three daily doses of injectable medications to select patients after the temporary pandemic guidelines concluded [47]. In a study of how take-home iOAT doses impacted patients’ quality of life and continuity of care, participants reported that take-home doses provided greater freedom, allowed them to establish daily routines, maintain privacy, engage in paid work, and manage their medication more independently [47]. There is also promising research on MMT take-home dosing during COVID-19 that showed patient benefits in terms of treatment retention [48]. As highlighted in this study, people who access iOAT experience structural vulnerabilities (e.g., insecure housing) that could prevent them from receiving a take-home dose. This study highlights the need to implement and evaluate iOAT take home doses, and the importance of additional research and guidance on how to develop take-home policies that are attentive to and work to address structural vulnerabilities to ensure equitable access. Notably, the documented benefits of take-home doses are particularly important in the context of recently changed policies in British Columbia requiring that all prescribed alternatives must be witnessed by a health professional [49].

Lastly, the increasingly toxic drug supply has posed challenges to iOAT program engagement and participants reported a need for greater choice in medication type including improved access to DAM and flexible approaches to doses. Other research on MMT programs have emphasized the need for modified dosing protocols given patients increased levels of opioid tolerance due to fentanyl use and suggests considerations regarding, initial dose titration rate, dose selection after missed dose days and take-home dose flexibilities [50]. Another study with iOAT service providers documented how providers negotiated a flexible approach to risk of intoxication for patients who are long term engaged with poly-substance use [39]. In our study, the ability to be responsive to ongoing drug use and the drug supply was facilitated by providers strong relationships with patients allowing for more flexible and individualized care including, the implementation of rapid dose titration schedules due to higher opioid tolerance from the toxic drug supply after periods of treatment disengagement. Study findings also point to the need to increase the availability of DAM and a wider variety of opioid treatment options and alternative medication formulation options that might improve people’s capacity to stabilize in the context of a worsening toxic drug supply. For instance, tablet hydromorphone treatment [26] as well as a fentanyl patch safer supply program [51] have been explored in our study setting. Overall, there is a need to ensure drug treatment programs are able to be responsive to the volatility of the drug supply to better meet the needs of patients.

## Limitations

This study involved participants from only four iOAT programs in Vancouver, BC, and may not be representative of other settings or regions. Second, while the sample included some diversity, certain groups, such as non-Indigenous people of colour and gender-diverse individuals, were underrepresented. While this analysis employed a structural vulnerability framework certain intersecting experiences of discrimination such as racism was not reflected in the study findings. However other research papers stemming from this data set that employed different conceptual frameworks highlighted findings about the experiences of Indigenous people and the experiences of women who access iOAT, highlighting how intersecting experiences of sexism and racism can impact treatment experiences (see for example, [37, 52]). Third, the COVID-19 pandemic hindered additional fieldwork and interviews, preventing a full exploration of participants' longitudinal experiences and limiting the scope of ethnographic observations. Finally, while data from different iOAT models was collected, the study did not aim to compare these models, and further research is needed in this area.

## Conclusion

This study highlights patients experiences and perceptions of facilitators and barriers to patient engagement in iOAT programs. Participants emphasized how access to a range of health and social services, relational care and flexible open-ended treatment delivery supported their engagement with treatment. However, barriers such as programmatic and drug policy-related rules (e.g., daily witnessed dosing and wait times), limited program capacity, and limited autonomy over medication formulation were identified as significant challenges, particularly for individuals with mobility challenges, housing instability and among patients seeking to balance treatment with employment. The study underscores the importance of flexible, patient-centered and equity-oriented care to meet the diverse needs of patients engaged in this treatment program.

## Data Availability

Relevant data are contained within the manuscript. Dataset cannot be made available for secondary analysis due to restrictions placed on data-sharing by the responsible research ethics board.

## Abbreviations

CAB: Community Advisory Board
CRA: Community Research Associate
DAM: Diacetylmorphine
HDM: Hydromorphone
iOAT: Injectable opioid agonist treatment
MMT: Methadone maintenance treatment
NAOMI: North American Opiate Medication Initiative
SALOME: Study to Assess Long-Term Opioid Medication Effectiveness
